# Exploration and Application of Malignant Cell Heterogeneity Analysis with Single-Cell Transcriptome Sequencing Technology

**DOI:** 10.14336/AD.2025.0836

**Published:** 2026-07-24

**Authors:** Wen Zhang, Tianliang Liu, Ting Liu, Yu Zhang, Zhenguo Zhao, Xinxin Zhang, Xiaoyang Li, Jiasheng Zhong, Zhicheng Li, Shifu Chen, Libin Xu

**Affiliations:** ^1^Department of immunology, National Cancer Center/National Clinical Research Center for Cancer/Cancer Hospital, Chinese Academy of Medical Sciences and Peking Union Medical College, Beijing, China.; ^2^Shenzhen Institute of Advanced Technology, Chinese Academy of Sciences, Shenzhen, China.; ^3^HaploX Biotechnology, Shenzhen, China.; ^4^LifeX Institute, Gannan Medical University, Ganzhou, China.; ^5^Department of Orthopedic Surgery, National Cancer Center/National Clinical Research Center for Cancer/Cancer Hospital, Chinese Academy of Medical Sciences and Peking Union Medical College, Beijing, China.

**Keywords:** scRNA-seq, Malignant cell, Heterogeneity of tumor

## Abstract

Cancer remains a formidable global health burden owing to its persistently high incidence and mortality, highlighting the need to elucidate its underlying biological mechanisms. Such insights are crucial for refining diagnostic precision and therapeutic efficacy. Notably, human tumors represent highly heterogeneous ecosystems, comprising a dynamic interplay between malignant cells and non-malignant components, such as immune infiltrates and stromal elements. This cellular heterogeneity constitutes a major obstacle in tumor research. Recent technological advancements have enabled the development and application of novel tools for investigating tumor heterogeneity. In this context, single-cell sequencing technologies emerged as transformative tools for dissecting tumor architecture at a cellular resolution, offering unprecedented insights into the cellular diversity and molecular underpinnings of cancer. In particular, single-cell RNA sequencing (scRNA-seq) has been widely used to analyze the tumor microenvironment, particularly its immune components, thereby providing a valuable framework for understanding tumor-immune system interactions. However, the intrinsic heterogeneity of tumor cells has received comparatively less attention, with limited studies focusing on these malignant populations. Comprehensive profiling of tumor cells has become increasingly feasible as scRNA-seq technologies continue to evolve, offering higher throughput, increased cell capture efficiency, and more advanced analytical pipelines. Despite these advancements, current applications of scRNA-seq remain primarily focused on immune and stromal cell populations. Therefore, a paradigm shift is warranted, redirecting the investigative focus toward tumor cells and gaining deeper insights into tumorigenesis and therapeutic vulnerabilities.

## 1. Cellular heterogeneity exists both within and between tumors

**Figure 1.**
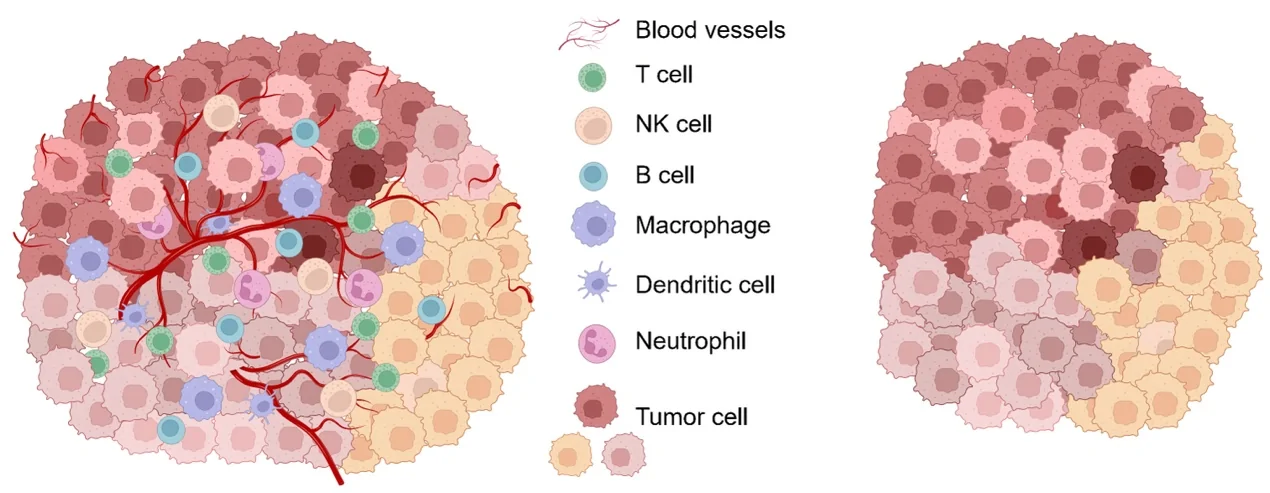
**Tumor heterogeneity at the microenvironmental and cellular levels**. This illustration depicts the two key dimensions of tumor heterogeneity. Left panel: The intratumoral microenvironment exhibits complex cellular diversity, including malignant tumor cells ( pink and red) and various stromal and immune cell types such as T cells (green), B cells (blue), natural killer (NK) cells (orange), macrophages (dark blue), dendritic cells (lavender), neutrophils (purple), and blood vessels (red), highlighting the spatial and cellular complexities of the tumor microenvironment (TME). Right panel: The tumor cell population demonstrates significant heterogeneity, visualized by the variable color intensity among tumor cells, representing distinct subclones or phenotypic states. This heterogeneity underlies diverse behaviors such as differential proliferation, invasiveness, and therapeutic resistance.

Tumor heterogeneity refers to the diversity at the cellular and molecular levels that exists within the same tumor, across different regions of the tumor, or among different individuals. This heterogeneity comprises variations in cell morphology, genotype, phenotype, and functionality. Tumor heterogeneity is a complex and significant concept in cancer research with profound implications for tumor development, treatment responses, and patient prognosis. Tumor heterogeneity, encompassing both inter-tumor variability across patients and intra-tumor diversity within individual neoplasms, is considered a hallmark of malignancy and a major barrier to effective cancer diagnosis, prognosis, and therapy [1, 2] (Fig. 1).

### 1.1 Inter-tumor heterogeneity

Inter-tumor heterogeneity demonstrates significant diversity among different types of tumors originating from various tissues and organs in the human body. Understanding the complexity of tumor heterogeneity is crucial for uncovering the mechanisms behind oncogenesis, disease progression, and therapeutic response [3-6]. This review explores the multifaceted nature of inter-tumor heterogeneity, spanning the genetic, epigenetic, and phenotypic dimensions, and highlighting its relevance to translational research and clinical decision-making across various cancer types.

#### 1.1.1 Genomic-Level Difference

Intertumor heterogeneity encompasses diverse genetic alterations that drive oncogenesis and tumor evolution across different cancer types. Genome-wide sequencing studies have revealed distinct mutational landscapes and driver mutations linked to specific tumor types, reflecting the tissue of origin, environmental exposure, and underlying genetic predisposition [7-12]. For example, TP53 mutations are commonly observed in various cancers, including breast [13, 14], lung [15, 16], and colorectal cancers [17, 18], whereas BRAF mutations are predominantly found in melanomas [19, 20] and certain thyroid cancers [18, 19]. Therefore, when testing tumors for mutations, particular attention should be given to the representative driver mutations unique to each cancer type. Similarly, molecular-targeted drugs designed for these mutations should be prioritized in clinical trials involving patients with the corresponding cancer types to improve treatment efficacy. Additionally, copy number variations (CNVs), chromosomal rearrangements, and structural variants contribute to inter-tumor genetic heterogeneity, shaping the clinical behavior and therapeutic vulnerabilities of various cancer types [20].

#### 1.1.2 Epigenetic-Level Difference

Epigenetic dysregulation, such as aberrant DNA methylation, histone modifications, and chromatin remodeling, is a pervasive feature across tumor types; however, substantial heterogeneity exists among different cancers. For example, in diffuse midline glioma, the loss of H3K27 trimethylation (H3K27me3) serves as a key oncogenic driver, promoting glial stem cell identity and silencing tumor suppressor genes. Conversely, elevated H3K27me3 levels in late-stage epithelial ovarian cancer enhance tumor angiogenesis and cell migration [21, 22].

In hematological malignancies, widespread DNA methylation aberrations, often driven by mutations in DNMT3A, TET2, or IDH1/2, occur in progenitor/hepatic cells, rendering these cancers responsive to DNMT and HDAC inhibitors [23]. Conversely, although solid tumors exhibit DNA methylation and histone modification abnormalities, these changes typically occur within differentiated cells and are highly heterogeneous. As a result, solid tumors often respond poorly to standalone epigenetic therapies such as demethylating agents or HDAC inhibitors.

#### 1.1.3 Phenotypic-Level Difference

Inter-tumor heterogeneity is evident at the phenotypic level, with diverse cellular morphologies, growth patterns, and metastatic behaviors observed across different cancer types. Histological classification schemes traditionally categorized tumors based on morphological features, such as glandular differentiation in adenocarcinomas [24, 25] and spindle-shaped cells in sarcomas [26-28]. However, recent advances in molecular profiling revealed additional layers of phenotypic diversity, including variations in cell signaling pathways, immune cell infiltration, and tumor microenvironment (TME) composition. For example, triple-negative breast cancers display distinct immune cell infiltrates and tumor-infiltrating lymphocyte subsets compared to hormone receptor-positive breast cancers, influencing their response to immunotherapy and targeted therapies [29-32].

#### 1.1.4 Metabolic-Level Difference

Different tumors exhibit distinct metabolic strategies, largely influenced by the tumor’s location and environment, which directly influence how tumor cells obtain energy and nutrients. For example, lung cancer cells often rely on glycolysis and oxidative phosphorylation due to the fluctuating oxygen and nutrients levels within their microenvironment [33, 34]. However, some lung tumors rely primarily on fatty acids for energy production [35, 36]. These metabolic differences affect tumor responses to drugs. In pancreatic cancer, the lack of oxygen and build-up of acidic conditions lead cancer cells to predominantly utilize glycolysis, reducing the efficacy of therapies such as EGFR or PD-1 inhibitors [37, 38]. Therefore, understanding the specific metabolic strategy of a tumor can help doctors select more effective treatments. For example, tumors that rely on fatty acid metabolism may benefit from a combination of fatty acid metabolism inhibitors and immunotherapies. Similarly, tumors that predominantly utilize glycolysis may respond more effectively to glycolysis-inhibiting drugs [39-41]. This metabolic approach could significantly improve cancer treatment. Diet plays a key role in shaping how tumors acquire energy and nutrients. However, different cancers respond differently to different dietary changes, and no single diet is effective across all malignancies. The ketogenic diet reduces the glucose supply to tumor cells, thereby forcing a shift towards less efficient energy-production methods and slowing tumor growth in gliomas and pancreatic cancers[39]. In contrast, a ketogenic diet low in soluble fiber increases levels of certain harmful bacteria, such as colibactin-producing E. coli, which can damage DNA and promote colonic inflammation [42]. Consequently, this diet may promote the growth of colon cancer.

#### 1.1.5 Microenvironment-Level Difference

The TME plays a pivotal role in shaping inter-tumor heterogeneity, as its cellular and molecular components vary substantially across cancer types. In pancreatic ductal adenocarcinoma (PDAC), the TME is characterized by extensive desmoplasia comprising myofibroblastic cancer-associated fibroblasts (CAFs), immunosuppressive macrophages, and sparse cytotoxic lymphocytes, which hinder therapeutic delivery and suppress anti-tumor immunity [43]. Single-cell RNA sequencing studies have revealed that PDAC harbors distinct CAF subpopulations with divergent functions: inflammatory CAFs that modulate immune responses, and myofibroblastic CAFs that remodel the extracellular matrix and promote chemoresistance [44]. In contrast, acral melanoma, a subtype prevalent in non-sun-exposed skin, exhibits a highly immunosuppressive micro-environment marked by reduced CD8^+^ T-cell infiltration, increased regulatory T-cells, and exhausted T-cell subsets expressing PD-1 and TIM-3 [45]. Moreover, these tumors exhibit increased chromosomal instability and upregulation of fatty acid metabolism pathways, which facilitate immune evasion and therapeutic resistance [46]. Such variability in stromal and immune architecture across cancers highlights the need for tumor-type-specific strategies targeting the microenvironment to overcome TME-driven resistance.

#### 1.1.6 Inter-Patient Heterogeneity Within the Same Tumor Type

In conclusion, intertumor heterogeneity is a complex and multifaceted phenomenon that reflects the intrinsic diversity of cancer biology. By elucidating the genetic, epigenetic, phenotypic, and microenvironmental factors underlying inter-tumor variability, researchers aim to develop novel therapeutic strategies that effectively target the distinct molecular vulnerabilities of individual tumors. Advancing our understanding of inter-tumor hetero-geneity and translating these insights into clinically actionable strategies requires the integration of multi-omics profiling, functional genomics, and systems biology approaches.

### 1.2 Intra-tumor heterogeneity

Tumors are rarely composed of a single, uniform population of cells. Instead, individual lesions often harbor a mosaic of subclones that differ in their genetic alterations, epigenetic states, and phenotypic traits. This internal diversity, known as intra-tumor heterogeneity, arises from the combined effects of somatic mutations, selective pressures within the tumor microenvironment, and continuous clonal evolution. Such variations can occur across different regions within the same tumor mass or even among adjacent cancer cells occupying a shared niche. Understanding the mechanisms underlying this heterogeneity is crucial for improving diagnostic accuracy and designing therapies that address tumor complexity. At the core of this phenomenon lies the accumulation of genetic alterations, including point mutations, chromosomal rearrangements, and genomic instability, that collectively drive clonal diversification. As tumor cells acquire mutations in oncogenes, tumor suppressor genes, and genes involved in DNA damage repair, they give rise to genetically distinct subclones that coexist and compete within the tumor microenvironment. Advances in high-throughput sequencing have enabled the systematic delineation of the mutational architecture of various cancers, revealing the layered contributions of driver mutations (e.g., KRAS mutations in colorectal cancer and EGFR mutations in lung adenocarcinoma), background passenger events, and cancer-type-specific mutational signatures (e.g., UV signature in melanoma, APOBEC signature in breast, and other cancers) [47-50]. Moreover, intra-tumoral genetic heterogeneity can be dynamic, with ongoing mutational processes shaping tumor evolution over time [51, 52].

Tumors exhibit spatial heterogeneity, characterized by distinct genetic, epigenetic, and phenotypic profiles across different regions of the tumor. This heterogeneity arises from variations in oxygen and nutrient availability, gradients of signaling molecules and growth factors, and interactions with the surrounding stroma[53-56]. For instance, hypoxic regions deep within a tumor may harbor cells with distinct metabolic adaptations and greater metastatic potential compared to well-oxygenated peripheral areas. Studies on renal cell carcinoma have demonstrated clear spatial segregation of driver mutations, such as VHL loss, across different tumor regions [57]. Imaging modalities, such as multi-regional sequencing, spatial transcriptomics, and mass spectrometry imaging, enable high-resolution spatial profiling of tumors, revealing spatially organized cellular ecosystems and niche-specific signaling networks [58-61]. Notably, spatial heterogeneity presents challenges for tumor sampling and diagnosis, given that molecular profiling based on a single tumor biopsy may fail to capture the full extent of intra-tumor heterogeneity.

Temporal heterogeneity captures the dynamic evolution of tumors throughout the disease, marked by the sequential accumulation of genetic mutations, epigenetic reprogramming, and phenotypic transitions in response to progression and therapeutic intervention. Longitudinal analyses have revealed the temporal dynamics of tumor evolution, uncovering patterns of clonal expansion, genetic diversification, and the emergence of therapy-resistant subpopulations driven by selective therapeutic pressures (e.g., BCR-ABL1 kinase domain mutations in CML under imatinib pressure and the EGFR T790M mutation in NSCLC under first-generation EGFR-TKI therapy) [62-65]. Moreover, tumor heterogeneity can be influenced by treatment-induced mutagenesis, immune-mediated selection, and microenvironmental changes, highlighting the need for adaptive treatment strategies that account for temporal variations in tumor biology [66-68].

Clonal evolution underlies the emergence of genetically distinct subclones within tumors and is driven by Darwinian selection, genetic drift, and clonal interference. Subclonal diversity contributes to intra-tumor heterogeneity by shaping tumor growth dynamics, metastatic dissemination, and therapeutic responses [62]. Recent advances in single-cell genomic technologies have enabled a detailed reconstruction of tumor clonal architecture, uncovering intricate phylogenetic relationships and spatially distinct subclonal populations with specialized functional characteristics [69-71]. Furthermore, intratumoral heterogeneity can influence treatment outcomes, as heterogeneous tumors may harbor subclones with distinct sensitivities to therapy (e.g., pre-existing minor subclones with BRAF mutations in melanoma resistant to BRAF inhibitors or subclones with altered drug metabolism), ultimately leading to treatment resistance and disease recurrence [72].

Intra-tumor heterogeneity presents major obstacles to accurate diagnosis, risk stratification, and therapeutic planning in oncology. Conventional histopathological evaluations often fail to capture the molecular and cellular complexities of tumors, underscoring the importance of integrative molecular profiling for individualized clinical management. Liquid biopsy technologies, which enable non-invasive detection of circulating tumor-derived DNA, RNA, and proteins, hold significant potential for real-time monitoring of tumor dynamics and predicting therapeutic responses [73]. Furthermore, evolutionary therapy, targeting the evolutionary vulnerabilities of tumors and preventing the emergence of resistant subclones, represents a promising avenue for addressing intra-tumor heterogeneity [74].

Intra-tumor heterogeneity represents a complex and dynamic feature of cancer that underlies biological variability and complicates clinical decision-making. By unraveling the genetic, epigenetic, and phenotypic landscapes of tumors, scientists aim to elucidate the underlying mechanisms driving tumor heterogeneity and identify novel therapeutic targets. Advancing our understanding of intra-tumor heterogeneity and translating these insights into personalized cancer therapies will require the integration of multiomic profiling, single-cell technologies, and spatially resolved approaches.

## 2. Single-cell transcriptome sequencing technology

The emergence of single-cell sequencing technologies can be traced back to the late twentieth century, followed by a series of progressive advancements in both experimental methodology and analytical frameworks. Over the past two decades, this field has undergone substantial evolution, marked by key innovations in technical platforms, refinement of protocols, and a dramatic expansion in biomedical applications. The following section outlines the major developmental phases and pivotal milestones that shaped the trajectory of single-cell sequencing.

Cells are the fundamental functional units of life. Each cell contains a wide array of biomolecules, including DNA, RNA, lipids, and proteins, as well as various nutrients and small molecules involved in metabolism. In the human body, trillions of cells coordinate with each other to perform various functions necessary for sustaining life. Cellular gene expression dictates a specific repertoire of RNA transcripts and proteins within cells. Although all somatic cells in the human body harbor an identical genomic sequence, differences arise through somatic mutations, epigenetic modifications, and context-dependent transcriptional regulation. These variations give rise to cell-type-specific profiles of membrane composition, ion channel expression, cytoskeletal architecture, growth factor signaling, receptor availability, and transcriptional activity.

Despite sharing an identical genome, cells within the same tissue exhibit remarkable functional diversity. This heterogeneity partly stems from the cell’s capacity to regulate its internal biochemical milieu, modulating concentrations of small molecules, RNA transcripts, and proteins within the cytoplasm and nucleus, thereby enabling a semi-autonomous control of cellular behavior. This semi-autonomous decision-making process can disrupt the inherent symmetry of multicellular organisms, arising from precise DNA replication. For example, cells differentiate by gradually acquiring specific intracellular molecules. Elucidating gene expression profiles at the single-cell level and uncovering the regulatory mechanisms governing transcriptional activity have long posed significant challenges in both biological research and clinical investigations.

**Figure 2.**
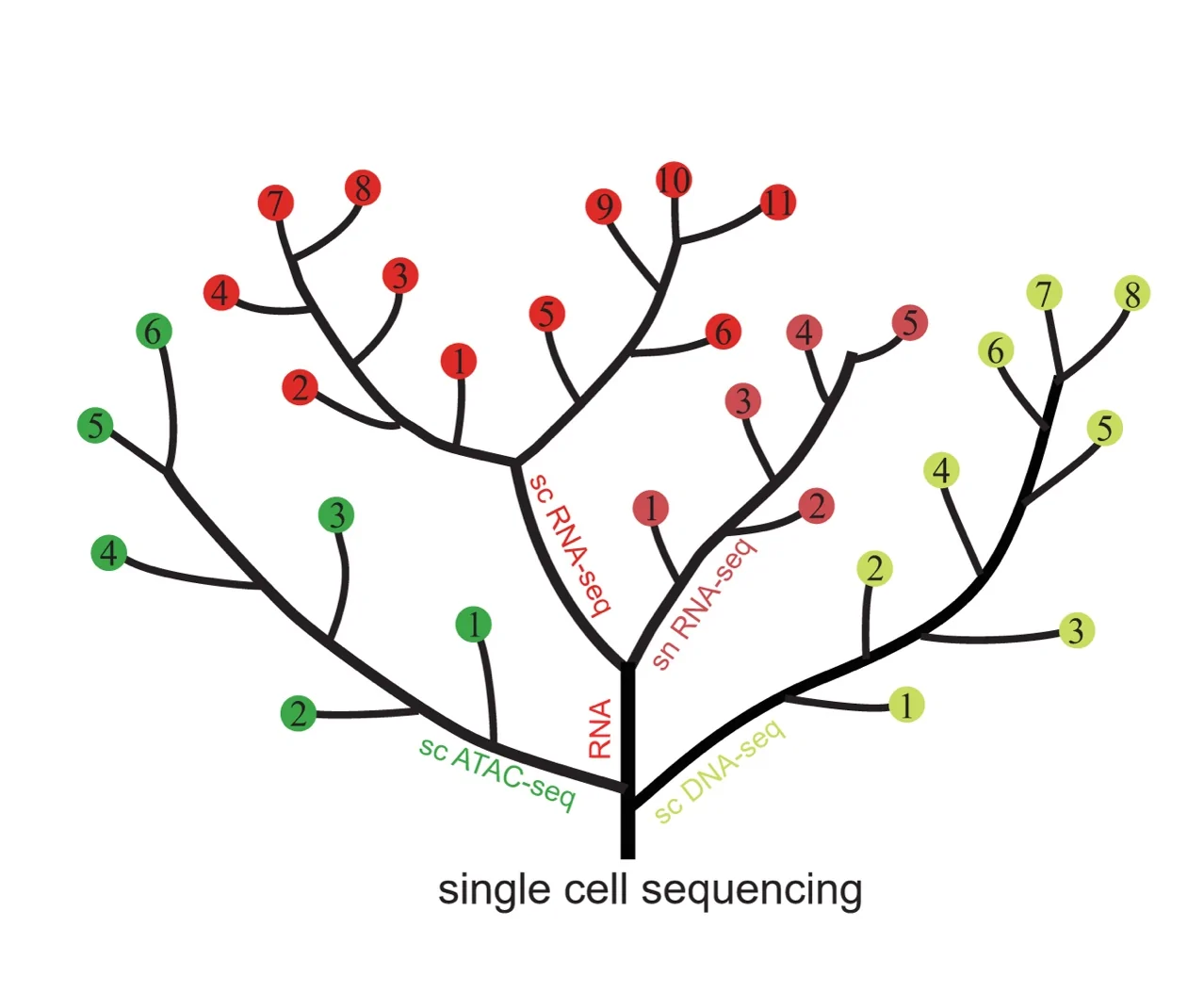
**Development and classification of single-cell sequencing technologies**. Single-cell sequencing has become a cornerstone of multi-omics research, with extensive applications in genomics, transcriptomics, and epigenomics

### 2.1 The development and applications of sequencing technology

Biotechnology has advanced continuously in parallel with rapid technological innovation. In 1977, Frederick Sanger introduced a DNA sequencing method called Sanger sequencing, based on the principle of chain termination induced by modified deoxyribonucleotides. This method earned Frederick Sanger the Nobel Prize in 1980. This marked the first time humans could interpret their genetic codes. However, despite their utility, early single-cell sequencing approaches were constrained by high costs and labor-intensive workflows. A pivotal advancement occurred in 2005 with the advent of next-generation sequencing (NGS), which significantly lowered sequencing expenses and facilitated high-throughput applications across a broad range of biological disciplines (Fig. 2).

However, due to the need for sufficient quantities of DNA or RNA, these studies were often limited to tissue-level analyses. For instance, the coexpression of all cells within a section of cancer tissue can offer insight into the internal immune status of a tumor. Tumor tissues comprise a complex mixture of malignant cells and various non-malignant mononuclear populations, including vascular endothelial cells, stromal fibroblasts, and diverse immune cell subsets. Consequently, bulk RNA sequencing analyses reflect only the average gene expression across all constituent cell types, thereby masking the cellular heterogeneity intrinsic to the tumor microenvironment. Bulk RNA sequencing limits cell type-specific gene expression; it only reflects the average expression of multiple cell types, potentially overlooking the roles of rare cells. Similar challenges are encountered when bulk RNA sequencing is used in disease-related research. Collecting the cells that comprise the tissue and analyzing gene expression information at a cellular level, including intra- and intercellular regulatory information, is crucial for addressing these issues.

An ideal method for identifying the composition of cell types within tissues or exploring the transcriptomic heterogeneity among cells of the same type, is to randomly extract individual cells from samples for separate analysis. Imaging-based approaches, such as single-molecule fluorescence in situ hybridization (smFISH), are constrained by their limited multiplexing capacity and typically allow the assessment of only a small number of target genes per cell [75] or flow cytometry. However, these methods cannot comprehensively capture cell transcriptome information. In contrast, scRNA-seq allows for a uniform and comprehensive capture of large quantities of mRNA within a single cell. The first scRNA-seq method was introduced in 2009 [76]. With continuous technological advancements, this method has steadily improved [77-82]. scRNA-seq has been widely applied in various areas, including the discovery of rare cell types [83] and investigation of tumor heterogeneity [84]. Navin et al. sequenced the DNA within individual cell nuclei, analyzed CNVs in the genome, and explored tumor development [85].

In addition to transcriptomic profiling, single-cell sequencing technologies have been extended to study epigenetic landscapes at single-cell resolution. A notable advancement in this domain was the development of a single-cell assay for transposase-accessible chromatin using sequencing (scATAC-seq) by Buenrostro et al., which leverages high-throughput, sequencing to map open chromatin regions and infer regulatory accessibility in individual cells (ATAC-seq) [86]. This method is currently widely used to explore open chromatin regions.

Single-cell sequencing enables rapid and precise characterization of thousands to millions of individual cells. Compared to bulk RNA sequencing, this method provides more accurate and detailed insights. For example, the scRNA-seq of cancer tissues can distinguish immune cells based on their gene expression characteristics, offering a more nuanced understanding than bulk RNA sequencing. Individual cell types can be further stratified into distinct functional subpopulations. T lymphocytes are typically classified as CD4^+^ helper T cells and CD8^+^ cytotoxic T cells, each exhibiting a specialized immunological role [70]. Single-cell sequencing has facilitated the discovery of previously unrecognized cell types and revealed the extent of cellular heterogeneity within complex tissues, highlighting its unparalleled resolution and transformative impact on cell population analysis [87]. The following sections outline the evolution and multifaceted applications of single-cell technologies across various molecular and cellular dimensions.

### 2.2 The development and applications of single-cell transcriptome sequencing technology

Recent advancements in single-cell sequencing technologies have enabled high-resolution analysis of individual cell genomes, transcriptomes, and epigenomes. A wide array of methods and applications has been developed to provide deeper insights into cellular heterogeneity and molecular regulation. The following sections summarize key developments and representative applications of single-cell genomics, transcriptomics, and epigenomics.

#### 2.2.1 Single-cell genomic sequencing technology

Genetic information is encoded on 46 chromosomes in human cells. During cell proliferation, whole-genome amplification begins from a single DNA molecule, which can lead to common issues, such as nucleotide loss and mutations. Variations occurring on chromosomes, such as Single-Nucleotide Variants (SNVs) and CNVs, can drive evolution and contribute to diseases such as cancer [88]. However, these DNA-level variations exhibit hetero-geneity within cell populations. To study these variations more precisely, it is essential to characterize the genome at the single-cell level [89-91].

Technological progress has driven the emergence of successive studies employing single-cell whole-genome sequencing, enabling the comprehensive analysis of genomic variation at single-cell resolution [92-96]. However, low genome coverage and high error rates pose challenges for single-cell whole-genome sequencing. These challenges hinder researchers’ ability to distinguish genuine genetic variations from errors caused by technical issues. Zong et al. addressed this challenge using a method involving multiple annealing and cycling amplification, achieving an increased genome sequencing coverage of up to 93%[97]. This method has been applied in studies mapping meiotic crossover sites [98]. Leung et al. developed a Single-Nucleus Exome Sequencing (SNES) method, further elevating genome coverage to 96% [99]. With the ongoing development of single-cell whole-genome sequencing technology, this method has found applications in diverse fields, including disease research [100, 101], prenatal diagnostics [102, 103], circulating tumor cell detection [104], and forensic analysis [105]. Despite these significant advances, the development of single-cell whole-genome DNA-sequencing methods still faces multiple challenges [106].

#### 2.2.2 Single-cell RNA sequencing technology

The quantification of transcripts allows for more accurate determination of cell states and molecular functions [107]. High-resolution RNA quantification provides more detailed and precise insights into the intracellular environment. scRNA-seq involves isolating and lysing individual cells, reverse-transcribing intracellular RNA into cDNA, and amplifying it to generate high-throughput sequencing libraries. Similar to single-cell whole-genome sequencing, scRNA-seq faces challenges in its development, particularly sensitivity, which refers to the probability of capturing and converting transcripts within a single cell into cDNA. Another challenge is ensuring that the quantification of scRNA-seq technology reflects the actual concentration of transcripts within the cell. Moreover, various scRNA-seq platforms vary in their strengths and limitations, each tailored to specific experimental objectives and analytical requirements. The earliest scRNA-seq method detected expression of only 5270 genes [76]. Subsequent improvements to scRNA-seq methods have emerged, including CEL-seq2, which employs a small-capacity automated microfluidic platform [108] and optimizes Smart-seq2 [80]. Library throughput can also be increased by incorporating barcodes into the primers [109, 110]. The addition of unique molecular identifiers (UMIs) to primers effectively distinguishes the original transcripts from randomly amplified ones [111], significantly improving the accuracy of scRNA-seq and has been applied to various sequencing methods [79, 109-112].

scRNA-seq has become a widely adopted and information-rich approach for constructing high-resolution cellular atlases across diverse tissues and even whole organisms [109, 113-117]. Owing to its unprecedented high resolution, scRNA-seq has been used to identify hundreds of previously unknown cell types across multiple species [118-124]. In addition to capturing molecular signatures within individual cells, scRNA-seq enables the investigation of molecular interactions across diverse cell types and organ systems, offering high spatial and temporal resolutions. Its translational potential has been increasingly recognized in clinical research. For instance, Velmeshev et al. conducted single-cell transcriptomic profiling of cortical tissues from individuals with autism spectrum disorder, revealing cell-type-specific transcriptional alterations implicated in disease pathophysiology [125]. This study identified distinct gene expression changes in the upper-layer projection neurons and selected glial subtypes linked to clinical severity in individuals with autism. Subsequent exome sequencing validated several of these genes as pathogenic variants linked to the disease etiology [126]. Similarly, Kim et al. conducted single-cell RNA sequencing on skin and peripheral blood samples from patients diagnosed with drug reactions with eosinophilia and systemic symptoms (DRESS), aiming to delineate the cellular and transcriptional alterations linked to this severe hypersensitivity condition [127]. Their analysis highlighted the JAK-STAT signaling pathway as a putative therapeutic target and demonstrated that pharmacological inhibition of JAK1 and JAK3 effectively mitigated disease-associated immune dysregulation.

Technological advancements have broadened the applicability of scRNA-seq. Owing to their simplicity and strong scalability, droplet-based technologies have become the most popular methods in this field. Despite significant refinements in the development of scRNA-seq, a common prerequisite across various sequencing methods is the use of high-quality single-cell or single-nucleus suspensions as the input. In cases involving fragile, oversized, or uneven quality tissues, the results of scRNA-seq may be suboptimal.

scRNA-seq primarily constructs libraries by reverse transcribing RNA from the cytoplasm and cell nucleus. However, when obtaining intact whole cells is not feasible, RNA extraction from the cell nucleus to construct snRNA-seq libraries is a viable alternative. Studies have demonstrated that nuclear mRNA content is sufficient to generate high-resolution transcriptomic profiles, enabling accurate identification of cell type-specific promoter usage [128, 129]. SnRNA-seq analysis of mouse neural progenitor cells revealed a high concordance between nuclear and whole-cell transcript levels. Notably, only a limited subset of genes, primarily those involved in mitochondrial respiration, exhibit differential abundance between the nuclear and cytoplasmic compartments [130]. snRNA-seq is a valuable alternative for profiling tumor cells, particularly in solid tumors, where conventional scRNA-seq is hindered by the large size and mechanical fragility of malignant cells during tissue dissociation [131-135]. Moreover, while scRNA-seq requires fresh and relatively intact samples, this requirement is not always met. Samples available for research are often frozen for extended periods. snRNA-seq circumvents this issue, thereby broadening the application of single-cell transcriptomics.

#### 2.2.3 Single-cell epigenetic sequencing technology

Advances in single-cell sequencing technologies have enabled epigenomic profiling at single-cell resolution, offering new insights into cellular identity and lineage specification. By investigating epigenetic features, such as DNA methylation patterns and chromatin accessibility, researchers can infer the developmental trajectory and differentiation status of individual cells. Single-cell bisulfite sequencing (scBC-seq) [136] and single-cell, single-base-resolution DNA methylation analyses based on reduced-representation bisulfite sequencing (scRRBS) [137] are effective methods for detecting DNA methylation at single-cell level.

Several powerful tools have been developed to detect cellular chromatin states, allowing the examination of protein modifications within individual cells. Rotem[138] introduced Single-cell Chromatin Immunoprecipitation followed by sequencing (scChIP-seq) based on droplet microfluidic technology. Using this technique, researchers have successfully mapped histone modification patterns, including those of H3K4me2 and H3K4me3, in mouse embryonic stem cells, embryonic fibroblasts, and hematopoietic progenitor cells, providing insights into cell-type-specific regulatory landscapes. Grosselin [139] analyzed the H3K27me3 landscape in patient-derived breast cancer xenografts using scChIP-seq, discovering unique H3K27me3 patterns in some sensitive tumors that differed from drug-resistant cells.

Genome chromatin accessibility can also be determined using high-throughput sequencing techniques such as ATAC-seq [86], which employs a hyperactive Tn5 transposase to insert sequencing adapters into accessible regions of the chromatin, enabling subsequent amplification and high-resolution identification of open chromatin landscapes. Currently, this technology can be applied to C1 and Cr systems, supporting scATAC-seq [140]. The C1 system utilizes integrated microfluidic technology to automate the entire library preparation workflow, including cell lysis, reverse transcription, and PCR amplification, thereby minimizing manual intervention and enhancing reproducibility [141]. In contrast, experimental protocols in the Chromium system require the isolation of nuclei and pretreatment with Tn5 transposase before droplet encapsulation and barcoding. scATAC-seq has been widely adopted to study transcriptional regulatory mechanisms in mixed cell populations across different lineages and developmental stages.

A growing repertoire of experimental and computational methodologies is continuously being developed to support the rapid evolution of single-cell sequencing technologies. Rapid advances in biotechnology now enable the extraction of extensive molecular information from individual cells that were previously challenging to access using traditional research methods, providing deeper insight into cell heterogeneity and rare cell populations. Over the past decade, the rapid evolution of single-cell sequencing has provided unprecedented insights into cellular diversity, lineage tracing, and the identification of rare cell populations. Nonetheless, despite substantial progress and a growing body of literature, many aspects of this dynamic field remain underexplored.

## 3. The application of single-cell transcriptome sequencing technology in tumor cells

scRNA-seq offers an unprecedented opportunity to resolve the functional and molecular heterogeneity of malignant tumor cells at a single-cell resolution. Unlike bulk RNA sequencing, which reflects the average gene expression across mixed cell populations, scRNA-seq enables precise delineation of malignant subclones, providing insights into clonal evolution, therapy resistance, and plasticity. These insights are now being consolidated in initiatives such as The Cancer Cell Atlas, which integrates large-scale scRNA-seq datasets across tumor types to map tumor cell states, transitions, and vulnerabilities [142, 143]. Incorporating clinical and pathological data with single-cell sequencing facilitates the discovery of novel diagnostic and prognostic biomarkers as well as the identification of disease-relevant cell types or cellular states that may inform therapeutic strategies [144]. In summary, The Cancer Cell Atlas serves as a valuable tool for guiding neoadjuvant therapy, particularly in scenarios lacking baseline guidance [145].

While scRNA-seq has significantly advanced the characterization of TME, an equally critical application lies in dissecting transcriptional heterogeneity within malignant cell populations. Tumors comprise diverse subclonal lineages with distinct expression programs that influence growth dynamics, drug resistance, immune evasion, and metastatic potential. For example, a pan-cancer scRNA-seq meta-analysis by Gavish et al. identified 41 recurrent “meta-programs” representing transcriptional states of malignant cells, including cell cycle, epithelial-mesenchymal transition (EMT), stress, and MYC-driven signatures, that recapitulate evolutionarily conserved hallmarks of intra-tumor heterogeneity (ITH) across 24 tumor types [146].

Guo et al. recently delineated a single-cell heterogeneity landscape in hepatocellular carcinoma (HCC) comprising three transcriptionally distinct tumor cell subtypes: an ARG1 metabolism subtype, TOP2A proliferative phenotype, and a S100A6^+^ EMT-like subtype [147]. These subtypes exhibit divergent biological programmes, lineage trajectories, and spatial distributions. Trajectory analysis revealed that both the metabolic and EMT subtypes evolved from a proliferative precursor, with EMT-like cells exhibiting stem-like properties and poor prognosis. Moreover, the EMT subtype also engaged in a positive feedback loop with cancer-associated fibroblasts through SPP1-CD44 and CCN2-TGFβ-TGFBR1 ligand-receptor axes, reinforcing metastasis and fibroblast activation. This study demonstrated how scRNA-seq can decode subclonal hierarchies and lineage plasticity with clinical implications.

In lung adenocarcinoma, spatially integrated single-cell profiling has revealed geographically segregated subclones with distinct EMT statuses and therapeutic resistance phenotypes [147]. These spatially defined subtypes were predominantly located in hypoxic niches and closely associated with fibroblasts or immune-excluded regions, illustrating the spatial constraints on subclonal behavior. Notably, such malignant programs often escape detection by bulk RNA sequencing methods. For instance, proliferative and EMT subtypes in HCC are indistinguishable using conventional bulk-defined classes, but are readily separated at single-cell resolution. These findings highlight the importance of high-resolution subclonal deconvolution in cancer biology.

In summary, single-cell transcriptomics enable the high-fidelity characterization of malignant subclonal diversity, stem-like tumor subsets, and evolutionary bottlenecks. This approach offers unique opportunities to reconstruct tumor phylogenies, identify high-risk phenotypes, and prioritize molecular targets for precision therapies. When combined with spatial, proteomic, or epigenetic data, scRNA-seq provides a comprehensive framework for decoding tumor evolution in situ.

The TME, comprising cellular and noncellular elements, plays a key role in cancer development, advancement, infiltration, spread, and medication resistance. These components shape the micro-environment that supports and hinders tumor growth, affects cancer progression, and contributes to tumor heterogeneity. The underlying molecular mechanisms of heterogeneity give rise to diverse malignant cell populations through genetic alterations, chromosomal rearrangements, epigenetic changes, and gene expression patterns. Unlike bulk RNA sequencing, which captures average gene expression and mutational profiles across heterogeneous cell populations, scRNA-seq allows for molecular characterization of individual cells. This resolution enables detailed mapping of the TME across various cancer types, including breast cancer [148]. scRNA-derived data offer critical insights that support refined molecular classification and inform the development of precise therapeutic strategies.

CAFs are prominent components of the tumor stroma and play a pivotal role in modulating the tumor microenvironment. Through excessive deposition of extracellular matrix (ECM) components and disruption of collagen crosslinking, CAFs alter tissue stiffness, promoting tumor progression and invasion [149-154]. For example, cadherin-11 promoted ECM accumulation, contributing to PDAC expansion and enhanced resistance to gemcitabine-based chemotherapy [155]. Moreover, CAF-derived factors modulate the immune micro-environment by suppressing immune effector cells and promoting the recruitment of immunosuppressive populations, ultimately enabling immune evasion by tumor cells [156-161]. Recent advances in single-cell transcriptomics have led to the identification of three major CAF subtypes: myofibroblastic CAFs (myCAFs), inflammatory CAFs (iCAFs), and antigen-presenting CAFs (apCAFs). Notably, a study demonstrated that stromal cells isolated from both primary cholangiocarcinomas and hepatic metastases exhibit highly conserved, patient-specific transcriptional signatures, suggesting that CAF phenotypes are predominantly shaped by intrinsic tumor-host interactions rather than tumor location [162, 163].

The immune microenvironment constitutes a critical component of the TME and plays a key role in modulating responses to immunotherapy and determining clinical outcomes. High-resolution immune profiling has revealed a dynamic landscape of immune cell populations, including lymphocytes, monocytes, macrophages, NK cells, and dendritic cells (DCs). As tumors progress, the immune system undergoes substantial remodeling, characterized by shifts in both stromal and immune cell subsets, collectively fostering an immunosuppressive milieu that supports tumor growth. For instance, during the progression of metastatic lung adenocarcinoma, resident myeloid cells are progressively replaced by monocyte-derived macrophages and DCs, a transition associated with T-cell exhaustion and diminished anti-tumor immunity. scRNA-seq has been employed to characterize distinct immune cell subsets within the tumor microenvironment, revealing preferential enrichment and potential clonal expansion of exhausted CD8^+^ T cells and regulatory T cells (Tregs) in HCC [70]. Moreover, the transcriptional signature of activated tumor-infiltrating Tregs, marked by elevated expression of genes such as *IL1R2*, has been associated with poor prognosis in lung adenocarcinoma [87].

Circulating tumor cells (CTCs) are rare malignant cells that detach from primary tumor sites and enter the peripheral bloodstream, where they are believed to act as “seeds” for metastatic dissemination and disease progression [164, 165]. Molecular profiling of CTCs at the single-cell level enables comprehensive genomic and transcriptomic datasets, providing valuable biomarkers for understanding the mechanisms underlying tumor progression, prognosis, disease dynamics, and therapeutic response [166-168]. The U.S. Food and Drug Administration (FDA) has approved CTCs and circulating tumor DNA (ctDNA) as non-invasive liquid biopsy biomarkers. Additionally, emerging analytes, such as extracellular vesicles (EVs), circulating tumor RNA (ctRNA), and tumor-educated platelets (TEPs), have gained recognition for their potential roles in cancer detection, prognosis, and therapeutic monitoring [167]. To overcome the challenges associated with CTC detection, such as low abundance, high background contamination by hematologic cells, cell loss during processing, and elevated false-positive rates, novel microfluidic and molecular platforms, such as ChemiXera X-iL20 and Hydro-Seq, have been developed to enhance sensitivity, specificity, and capture efficiency [169, 170].

Evidence suggests that CTCs tend to travel in groups, rather than individually, in the bloodstream [171]. Characterizing the phenotypic diversity of CTCs and their interactions with circulating immune cells provides key insights into the mechanisms driving tumor progression and prognostication. In breast cancer, distinct CTC subpopulations have been identified, varying in their estrogen receptor responsiveness, degree of EMT, and capacity to engage in crosstalk with monocytes in the peripheral blood [172]. Furthermore, the release of transforming growth factor-β (TGF-β) by activated platelets has been implicated in inducing EMT-like phenotypes in CTCs, thereby facilitating their escape from immune surveillance [165]. Moreover, a scRNA-seq study revealed that CTCs reside within a distinct neutrophil-associated niche in the peripheral circulation. Neutrophils in this microenvironment exhibited elevated expression of genes involved in cell proliferation and pro-inflammatory cytokines, including *TNF-β*, *OSM*, *IL-1β*, *IL-6*, and *VCAM1*, indicating their active role in promoting CTC survival and metastatic potential [173]. This finding indicates that CTC-neutrophil clusters may serve as clinically relevant biomarkers for disease progression, providing novel insights that could inform personalized therapeutic strategies for cancer management. Notably, Velpula and Buddolla (2025) demonstrated that integrating nanotechnology-based enrichment platforms with scRNA-seq significantly improved CTC detection and characterization [174]. Their study identified molecular signatures associated with pro-metastatic gene expression (e.g., *MMP9*, *CXCR4*, *FN1*) and highlighted rare subclones with the potential for dormancy and late relapse. These insights highlight the clinical value of scRNA-seq for tracking minimal residual disease and informing anti-metastatic therapies.

Recent advances in spatial transcriptomics (ST) significantly complemented scRNA-seq in resolving spatially distinct transcriptional programs of malignant tumor cells within the tissue context. Unlike scRNA-seq, which requires tissue dissociation, thereby losing positional information, ST enables the mapping of gene expression back to its original spatial coordinates, facilitating the identification of spatial niches occupied by tumor subclones. For instance, Wang et al. (2025) conducted integrated single-cell and spatial transcriptomic profiling on colorectal cancer tissues, revealing spatially segregated tumor cell subtypes with different degrees of EMT and therapy resistance. These subtypes show differential proximity to fibroblasts and immune-infiltrated regions, suggesting that micro-environmental factors influence intratumoral heterogeneity [175]. Similarly, Qiu et al. (2025) identified GREM1^+^ fibroblast clusters spatially co-localized with SPP1^+^ macrophages in gastric cancer using multi-omics integration, demonstrating that malignant cell states are not solely molecularly diverse but also functionally stratified based on spatial micro-niches, affecting treatment responsiveness and prognosis [176]. Moreover, spatial transcriptomic reconstruction has helped delineate hypoxia-driven regional transcriptional programs in glioblastomas, revealing that malignant cells in hypoxic zones exhibit an elevated expression of angiogenic and metabolic adaptation genes, contributing to locoregional therapy resistance [177]. These findings underscore the necessity of integrating spatial information with single-cell- resolution data to fully understand the context-specific heterogeneity of malignant tumor cells, which holds critical implications for biomarker discovery and targeted therapy development.

## 4. Limitations and Future Directions

### 4.1 Limitations

scRNA-seq has revolutionized the study of tumor heterogeneity. However, several core technical limitations remain unresolved, constraining interpretability and clinical application. One major limitation is the dropout effect, characterized by the probabilistic loss of transcripts from lowly expressed genes during capture and library preparation, resulting in sparse expression matrices and the underestimation of key regulatory genes, especially in rare cell types [178]. Another persistent limitation is dissociation bias. Most scRNA-seq protocols require enzymatic digestion of solid tissues, a step that can cause the selective loss of fragile, large, or adherent cell populations, as well as induce stress response signatures that confound the biological interpretations [179]. This artifact is particularly problematic in tumor samples where the epithelial and stromal compartments differ in resilience. Additionally, Batch effects further complicate the data interpretation. Variations in sample handling, library protocols, or sequencing depth can introduce artificial clusters, posing a major barrier to data integration between patients or studies[180]. Moreover, the presence of doublets, whereby multiple cells were captured together, remains a confounding factor that can distort the clustering results and affect lineage inference [181].

Computational and experimental strategies have been developed to address these issues. Imputation algorithms such as MAGIC, SAVER, and scImpute, estimate missing expression values to mitigate dropout-associated noise [182-184]. While useful, these tools can oversmooth the data and obscure true biological variability. Batch correction methods, including Harmony, mutual nearest neighbors’ correction, and Seurat v3 integration allow the alignment of datasets from multiple sources. These algorithms have demonstrated good performance in harmonizing data across platforms and tumor types [180, 185]. Doublet detection tools such as DoubletFinder and Scrublet reduce false positives during clustering and cell-type annotation [181, 186]. However, current doublet detection methods remain insufficiently reliable for rare or transcriptionally similar populations. In addition to computational solutions, multimodal strategies were implemented. ST platforms, including 10x Visium and Slide-seq, restore the spatial context lost during tissue dissociation [187, 188]. For instance, spatial mapping of malignant clones in glioblastoma revealed subclonal topographies that were not detectable by scRNA-seq alone [189]. Integration of single-cell proteomic and epigenomic data further enhanced cell-state resolution. Technologies such as CITE-seq and ASAP-seq simultaneously measure surface proteins or chromatin accessibility along with transcriptomes, enabling more refined functional annotation of tumor-infiltrating immune cells [190, 191].

Collectively, these approaches address several key limitations of scRNA-seq. However, several challenges remain to be resolved. High dropout rates, dissociation-induced artifacts, and integration difficulties limit the full resolution of tumor subclonality. Continuous improvements in experimental protocols, statistical modeling, and cross-platform integration remain essential.

### 4.2 Lineage Tracing

Despite the advances driven by scRNA-seq, this technique only provides a static snapshot of cellular states and cannot capture ancestral relationships, fate decisions, or the temporal dynamics underlying tumor progression and therapeutic adaptation, thereby limiting our understanding of how malignant subpopulations evolve, interact, and respond to treatments.

To address these limitations, lineage-tracing technologies have been developed to resolve clonal dynamics over time. These methods involve labeling cells with heritable markers, either artificially (e.g., lentiviral barcodes or CRISPR-Cas9 scars) or by leveraging naturally occurring somatic mutations such as mitochondrial DNA variants or nuclear SNVs. These marks are transmitted to daughter cells, enabling the reconstruction of clonal trees and developmental trajectories, particularly when combined with transcriptomic or epigenomic data. A landmark study by Quinn et al. used CRISPR-based barcoding (scGESTALT) in a zebrafish melanoma model to track the emergence of metastatic clones [192]. The study revealed that early-emerging clones, rather than proliferative ones, dominated distant metastases, an insight unobtainable from scRNA-seq alone. Similarly, Al'Khafaji et al. applied endogenous mitochondrial variant-based tracing in human AML and revealed that relapse arises not from the dominant clone at diagnosis, but from a minor, pre-existing stem-like clone that escaped therapy [193].

These findings highlight the unique strength of lineage tracing, which allows researchers to move beyond correlation-based inferences (e.g., transcriptomic similarity) and uncover causal clonal relationships critical for understanding tumor plasticity, relapse, and therapy resistance. Importantly, lineage tracing helps resolve fundamental questions in tumor biology: Which subclones give rise to invasive or metastatic populations? Do drug-resistant phenotypes arise from selection or transdifferentiation, and do multiple phenotypically distinct populations originate from a common cancer stem-like ancestor? These capabilities extend beyond mechanistic insights and carry direct clinical relevance. For instance, identifying early relapse-competent subclones offers opportunities to design preemptive combination therapies aimed at eliminating them before they expand. Nonetheless, lineage tracing approaches also face limitations. Barcode-based systems (e.g., GESTALT and Polylox) are mostly applicable to model organisms and require genetic manipulation. Endogenous mutation-based strategies (e.g., mtDNA and SNV phylogenies) can be applied to human samples but offer limited resolution and may introduce noise. Moreover, most lineage-tracing techniques lack spatial context, limiting the ability to link clonal identity with local microenvironmental interactions.

Recent efforts have aimed to overcome these gaps by integrating lineage information with spatial transcriptomic or proteomic mapping. For example, Leeper et al. introduced an approach that combines natural mutation tracing with spatial transcriptomics to reconstruct the topography of clonal evolution within tumor tissue sections [194]. These innovations point toward a future in which lineage tracing becomes a standard complement to scRNA-seq, enabling dynamic, spatially resolved, and mechanistically grounded models of tumor evolution. Rather than simply describing cell states, lineage tracing allows us to understand how these states arise, persist, or disappear during therapy, making it a critical tool for advancing precision oncology.

In summary, while single-cell and spatial technologies have already begun to reshape our understanding of malignant tumor heterogeneity, significant technological, analytical, and clinical challenges remain. Addressing these limitations will be crucial to translating high-resolution insights into actionable precision oncology.

### Conclusion

The heterogeneity of malignant cells within tumors presents a significant challenge in cancer research and treatment. Historically, the variability among cancer cells within a single tumor has hindered the development of universally applicable findings, resulting in a limited focus on individual differences among malignant cells. This cellular diversity can lead to varying responses to treatments, thereby affecting the effectiveness of therapeutic interventions.

However, recent technological advancements have opened new avenues for more precise and individualized research. One major technological advancement is scRNA-seq, which enables high-resolution profiling of transcriptomic landscapes at the cellular level of individual cells. This technique is particularly valuable in cancer research for several reasons. scRNA-seq enables the resolution of cellular heterogeneity within tumors by identifying discrete subpopulations, including rare cells that may exhibit resistance to conventional therapies. Characterization of these subclones provides critical insights for the development of more precise and effective treatment strategies. In contrast, bulk RNA sequencing averages transcriptomic signals across mixed cell populations, potentially obscuring the presence and biological relevance of minor but clinically important subsets such as cancer stem cells. Single-cell techniques can be used to identify these rare cell types and their unique characteristics. Moreover, by analyzing tumor cells at different stages or under different treatment conditions, researchers can track the evolution and adaptation of tumors over time. This information is essential to elucidate the mechanisms underlying therapeutic resistance and metastatic progression.

The application of scRNA-seq in cancer research is still in its early stages for many types of cancer. While substantial insights have been gained from studying a small number of malignancies such as breast cancer and melanoma, many other cancer types have yet to be extensively studied using this technology. These limitations stem from the substantial cost associated with scRNA-seq, the computational complexity of downstream data processing, and the requirement for specialized analytical expertise to ensure accurate interpretation of the results.

Increasing the accessibility and affordability of scRNA-seq, improving computational tools for data analysis, and fostering collaboration across disciplines are essential to expanding the use of this powerful technique in cancer research. The ultimate goal is to leverage the detailed insights offered by scRNA-seq to develop more precise and personalized cancer therapies, thereby enhancing patient outcomes across diverse cancer types.
